# Comparative study of dinoprostone and misoprostol for induction of labor in patients with premature rupture of membranes after 35 weeks

**DOI:** 10.1038/s41598-022-18948-5

**Published:** 2022-09-02

**Authors:** Flavie Sire, Laure Ponthier, Jean-Luc Eyraud, Cyrille Catalan, Yves Aubard, Perrine Coste Mazeau

**Affiliations:** 1 Department of Gynaecology and Obstetrics, Mother and Children’s Hospital, Limoges Regional University Hospital, 8 Avenue Dominique Larrey, 87000 Limoges, France; 2 Department of Pediatrics, Mother and Children’s Hospital, Limoges Regional University Hospital, 8 Avenue Dominique Larrey, 87000 Limoges, France; 3 Centre de Biologie et de Recherche en Santé, CHRU Limoges, Université de Limoges, Inserm U1092, 2 rue du Pr Bernard Descottes, 87000 Limoges, France

**Keywords:** Health care, Medical research

## Abstract

The modalities of induction of labor in the event of premature rupture of membranes are controversial. The main purpose of this study was to compare the modalities of delivery after the use of dinoprostone or misoprostol for labor induction in the preterm rupture of membranes after 35 weeks in women with an unfavorable cervix. We then studied maternal and fetal morbidity for the two drugs. Retrospective, single-center, comparative cohort study in a level 3 maternity unit in France from 2009 to 2018 comparing vaginal administration of misoprostol 50 µg every six hours (maximum 150 µg) and administration of dinoprostone 10 mg, a slow-release vaginal insert, for 24 h (maximum 20 mg), for labor induction in the preterm rupture of membranes after 35 weeks in women with an unfavorable cervix (Bishop score < 6). We included 904 patients, 656 in the misoprostol group and 248 in the dinoprostone group. Vaginal delivery rate was significantly higher in the dinoprostone group (89% vs. 82%, *p* = 0.016). There were more cesarean sections for abnormal fetal heart rate in the misoprostol group (*p* = 0.005). The time interval from induction to the beginning of the active phase of labor and the duration of labor were shorter in the misoprostol group than in the dinoprostone group (437 min vs. 719 min, *p* < 0.001 and 335 min vs. 381 min, *p* = 0.0023, respectively). Maternal and neonatal outcomes were not significantly different in the two groups. Vaginal dinoprostone used for labor induction in preterm rupture of membranes seems to be more effective for vaginal delivery than vaginal misoprostol (50 µg).

## Introduction

Premature rupture of membranes (PROM) is a frequent obstetric event which occurs in about 8–10% of pregnancies; about 60% of these cases are term pregnancies^1^. Rupture of membranes before 37 weeks of gestation (WG) is named preterm premature rupture of membranes (PPROM) and occurs in about 2–3% of pregnancies before 34 WG^2^. To date, the modalities of induction of labor in the event of PROM are controversial in terms of timing but also of which drugs to use^3–6^.

If the cervix is considered favorable, the agent most used is oxytocin, which is administered intravenously to induce labor. If the cervix is unfavorable (Bishop score < 6), prostaglandin E2 and misoprostol are frequently used to induce cervical ripening.

Following media coverage in France of serious adverse effects with Cytotec (misoprostol), including in off-label use, the National College of French Obstetricians Gynecologists (CNGOF) issued a press release in October 2017 prohibiting its use in obstetrics ^7^. Pfizer withdrew Cytotec from the market in some countries and in particular in France in March 2018.

In January 2018, Angusta 25 µg (misoprostol), which was already widely used in some countries, was granted a marketing authorization (MA) in France for use in obstetrics in induction of labor.

The main purpose of this study was to determine the modalities of delivery in patients with PROM after 35 WG, comparing the use of misoprostol 50 µg (Cytotec) and dinoprostone 10 mg (Propess) vaginally in the absence of spontaneous labor. We also recorded in the two groups the time from induction to the beginning of the active phase of labor and the duration of labor and studied whether the type of drug used affects maternal and fetal morbidity and mortality.

## Material and methods

This retrospective, comparative, single-center study was conducted in a level 3 maternity unit (2600 deliveries per year) in the Gynecology and Obstetrics Unit of the Limoges Mother and Child Hospital (HME), in France, from 2009 to 2018. Patients in whom labor was induced by Cytotec were included from 2011 to 2017 and those administered Propess were included from 2009 to 2018.

This study complied with the Helsinki guidelines for human research and was approved by the Limoges Regional University Hospital institutional review board (297-2019-63) on 10 April 2019.

Inclusion criteria were singleton pregnancies with a cephalic presentation and induction of labor by Cytotec or Propess after 35 WG and with an unfavorable cervix (Bishop score < 6) in the case of premature rupture of membranes. Twin pregnancies, fetuses in the breech or transverse position, uterine scarring, term < 35 WG, in utero fetal death and medical termination of pregnancy were excluded, as were patients with a favorable cervix (Bishop score ≥ 6) or those who received the 2 drugs (dinoprostone and misoprostol) or a third for induction of labor.

The diagnosis of PROM was made either clinically or using an Insulin-like Growth Factor-Binding Protein-1 diagnostic test (IGFBP-1) (Actim PROM or Amniodiag). After 35 WG, according to our departmental protocol, induction of labor was initiated within 6 h of rupture in cases where a vaginal swab (VS) was positive for Streptococcus B, or within 12 h of rupture in cases where the VS was Streptococcus B negative. When the VS result was unknown, induction of labor was started within 6 h of rupture, as in patients with Streptococcus B positive.

Before October 2017, misoprostol 200 µg (Cytotec) was used to induce labor vaginally (one-quarter of a tablet, i.e., 50 µg), which was renewed every 6 h in the absence of sufficient cervical modification (Bishop score < 6). The maximum dose administered was 150 µg or three-quarters of a tablet.

From October 2017, when Cytotec was no longer available in France, induction of labor was started using vaginal dinoprostone 10 mg (Propess), for a period of 24 h, after which, if the cervix was still unfavorable (Bishop score < 6), a second dinoprostone vaginal insert was administered. Before 2017, a small number of obstetricians from our team preferred to start the induction of the labor by the use of Propess. Oxytocin was employed to induce labor if the cervix was considered favorable (Bishop score ≥ 6).

In the cervix remained unfavorable after the administration of 150 µg misoprostol or after two dinoprostone vaginal inserts, induction of labor was continued using oxytocin (Syntocinon): 5 IU of oxytocin was diluted in 49 mL of physiological saline and was started at 2 mIU/min, a flow rate of 1.2 mL/h with an increase of 2 mIU every 30 min up to 12 mL/h or until uterine contractions were sufficient.

During induction of labor, fetal heart rate was monitored 30 min before and 2 h after placement of Propess or Cytotect and then monitored for 30 min minimum every 6 h.

Antibiotic coverage was provided by amoxicillin in the absence of allergy, using the same protocol for both groups.

Vaginal delivery rate was our primary outcome. The secondary outcomes were:

- comparison of induction failure [absence of labor after placement of one or two dinoprostone vaginal inserts or after 150 µg of misoprostol (cervical dilatation still < 3 cm)];
- time from induction to the beginning of the active phase of labor (cervical dilatation ≥ 3 cm);
- duration of labor (time in minutes from attainment of 3 cm cervical dilatation to delivery);
- maternal and fetal morbidity and mortality.

Fetal heart rate was continuously recorded during labor. Abnormal fetal heart rate was classified as suspicious or pathological according to the FIGO classification (International Federation of Gynecology and Obstetrics)^8^. We also recorded fetal heart rate abnormalities that required monitoring by lactate sampling at the fetal scalp or in connection with abnormal uterine contractility. Uterine hypertonicity was defined as the occurrence of a prolonged decrease in fetal heart rate requiring the administration of nitrate (Risordan). Uterine tachysystole was defined as the occurrence of more than five uterine contractions per 10 min.

Data were analyzed using JMP 14.2.0 software (SAS Institute, Cary, USA). Descriptive statistics of quantitative data were reported as mean ± standard deviation and compared with the use of Student's t-test. The confidence interval was 95%. Qualitative data were presented by numbers and percentages and compared using a Chi-square or Fisher’s exact test according to the conditions of application. *p* values < 0.05 were considered statistically significant.

### Ethics approval

Research involving human subjects complied with all relevant national regulations, institutional policies and is in accordance with the tenets of the Helsinki Declaration (as revised in 2013), and was approved by the Limoges Regional University Hospital institutional review board, the local ethics committee (297-2019-63) on April 10, 2019.

### Informed consent

Informed consent was obtained from all individual participants included in the study.

## Results

Of 904 patients included in this study, 248 were in the dinoprostone group and 656 in the misoprostol group. Patient characteristics are listed in Table 1. The average age was 29.43 years (± 0.22) in the misoprostol group and 30.33 years (± 0.38) in the dinoprostone group, which was statistically significantly different (*p* = 0.03), but not clinically relevant insofar as the average age of the two groups was less than 40 years, a criterion known to influence the data we studied. There was no significant difference in the other criteria that could affect our primary outcome.

**Table 1:** Patient characteristics.

Patient characteristics	Dinoprostone	Misoprostol	*p*
	N = 248	N = 656	
	Mean ± SD	Mean ± SD	
	n (%)	n (%)	
**Age**	30.33 ± 0.38	29.43 ± 0.22	***0.0332***
≥ 40 years	8 (3.23)	21 (3.20)	0.98
**Parity**	1.58 ± 0.06	1.48 ± 0.04	0.160
Primiparity	161 (64.9)	453 (69.1)	0.23
Multiparity (≥ 2 children)	87 (35.08)	203 (30.95)	0.23
**Body mass index**	24.6 ± 0.33	24.15 ± 0.20	0.215
**WG at time of rupture**	39.28 ± 0.11	39.22 ± 0.06	0.609
WG at time of rupture: median (interquartile range)	39.42 (± 2.67)	39.42 (± 2)	0.3
**Rupture < 37 WG**	31 (12.5)	61 (9.3)	0.163
WG at time of rupture	35.86 ± 0.11	35.88 ± 0.07	0.95
WG at time of rupture: median (interquartile range)	35.85 (1.14)	36 (1.14)	0.95
**VS Streptococcus B + **	35 (14.1)	92 (14.0)	0.147

VS = Vaginal swab; WG = weeks of gestation. Significant values are in bolditalics.

### Mode of delivery

The rate of vaginal delivery in the dinoprostone group was statistically higher (*p* = 0.016) than in the misoprostol group (Table 2). We found no statistically significant difference between the two groups for instrumental deliveries. The rate of cesarean delivery for abnormal fetal heart rate was significantly higher (*p* = 0.005) in the misoprostol group (Table 2).

**Table 2:** Mode of delivery and cesarean indications.

	Dinoprostone	Misoprostol	*p*
	N = 248	N = 656	
	n (%)	n (%)	
**Mode of delivery**			***0.016***
Cesarean	28 (11.3)	116 (17.7)	
Vaginal	220 (88.7)	540 (82.3)	
**Vaginal delivery**			
Normal	177 (71.4)	406 (61.9)	***0.007***
Instrumental	43 (17.34)	134 (20.4)	0.29
**Reasons for cesarean**			NS
Abnormal fetal heart rate	14 (50)	76 (65.5)	***0.005***
Failed induction	4 (14.29)	6 (5.17)	NS
Arrest of dilatation	5 (17.86)	21 (18.1)	NS
Arrest of descent	5 (3.52)	9 (7.76)	NS
Other (cord prolapse, hemorrhage…)	0 (0)	4 (3.45)	NS

NS = Non-significant. Significant values are in bolditalics.

In our sub-group analysis, there was no significant difference between the two groups in fetal acidosis (umbilical arterial pH < 7.15 and lactate levels > 5 mmol/L), for the patients who had a cesarean for abnormal fetal heart rate (2/14 or 14.3% of the newborns in the dinoprostone group vs 15/76 or 19.7% in the misoprostol group; with *p* = 0.69).

For the induction of labor before 37 WG, there were 31 patients in the dinoprostone group (12.5%) versus 61 patients in the misoprostol group (9.3%) (*p* = 0.163). There was no significant difference between the two groups in terms of the mode of delivery (Table 3).

**Table 3:** Mode of delivery before 37 weeks of gestation.

Delivery	Dinoprostone	Misoprostol	*p*
	N = 31	N = 61	
	n (%)	n (%)	
Vaginal delivery	28 (90.32)	56 (91.80)	0.81
Cesarean section	3 (9.68)	5 (8.20)	

### Time from induction to the onset of labor/duration of active labor

The time from induction to the onset of labor was 11 h 58 min (718 min) in the dinoprostone group and 7 h 17 min (437 min) (718 vs. 437; *p* < 0.001) in the misoprostol group, so labor started more quickly in the misoprostol group (Table 4). The duration of active labor was 6 h 21 min (381 min) in the dinoprostone group and 5 h 34 min (334 min) in the misoprostol group. Patients who had induction with misoprostol gave birth 47 min earlier than those who had induction with dinoprostone (*p* = 0.0023).

**Table 4:** Comparison of interval from induction to the onset of labor and duration of labor.

Duration (min)	Dinoprostone	Misoprostol	*p*
	N = 248	N = 656	
	n (%)	n (%)	
Interval from induction to the onset of labor	718.29 ± 29.32	436.69 ± 18.12	**< *0.001***
Labor	381.4 ± 13.26	333.67 ± 8.21	***0.0023***

Significant values are in bolditalics.

### Abnormal fetal heart during labor

There was no significant between-group difference in abnormal fetal heart rate (*p* = 0.058) (Table 5). There was more tachysystole in the misoprostol group, though the difference was not statistically significant (*p* = 0.45), but there was significatively more uterine hypertonicity in the dinoprostone group (*p* = 0.025) (Table 5).

**Table 5:** Fetal heart rate abnormalities during labor.

	Dinoprostone	Misoprostol	*p*
	N = 248	N = 656	
	n (%)	n (%)	
AFHR	73 (29.44)	229 (34.91)	***0.058***
Tachysystole/hypertonia/lactate	39 (15.73)	116 (7.68)	NS
• Tachysystole	8 (20.5)	30 (25.86)	0.45
• Hypertonia	16 (41.0)	26 (22.41)	***0.025***
• Lactate	15 (38.5)	60 (51.72)	0.29
Suspected or pathological AFHR (FIGO)	34 (13.71)	113 (17.23)	NS

AFHR: Abnormal fetal heart rate.

FIGO: International Federation of Gynecology and Obstetrics. Significant values are in bolditalics.

### Maternal morbidity

The dose of misoprostol or dinoprostone was not the same for all patients. In the dinoprostone group, one 10 mg dinoprostone vaginal insert was generally used, though in some cases 2 or even 3 were used in exceptional cases of loss of a vaginal insert by the patient. In the misoprostol group, one-quarter of a 200 µg misoprostol tablet was generally used, i.e., 50 µg, though in some cases two- or three-quarters of a 200 µg misoprostol tablet were administered.

There was no difference between the two groups in the mode of delivery depending on the dose of misoprostol or dinoprostone (Table 6).

**Table 6:** Dose of dinoprostone and misoprostol and mode of delivery.

Dose	Dinoprostone 10 mg	Misoprostol 50 µg	*p*
	N = 248	N = 656	
	n (%)	n (%)	
1	227 (91.53)	567 (86.43)	
Vaginal delivery	202 (88.98)	471 (83.07)	0.26
Cesarean section	25 (11.01)	96 (16.9)	0.11
≥ 2	21 (8.47)	89 (13.57)	
Vaginal delivery	18 (85.7)	69 (77.52)	0.17
Cesarean section	3 (14.28)	20 (22)	0.12

There was no significant between-group difference in hemorrhaging during delivery (> 500 mL; 7 in the dinoprostone group (2.85%) and 24 in the misoprostol group (3.89%) *p* = 0.55) or in treatment if there were delivery complications (with or without hemorrhage). There were no cases of vascular ligation or hemostatic hysterectomy.

In the post-partum period, there was one maternal infection in the dinoprostone group (0.40%) and six in the misoprostol group (0.91%). The difference was not statistically significant.

The duration of maternal hospitalization after delivery was 4.13 days (3.993–4.266) in the dinoprostone group and 4.35 days (4.271–4.438) in the misoprostol group (*p* = 0.006).

There were no cases of uterine rupture, transfer to intensive care, or maternal death.

### Neonatal outcomes

Neonatal characteristics and neonatal morbidity are reported in Table 7.

**Table 7:** Characteristics of newborns and neonatal morbidity.

	Dinoprostone	Misoprostol	*p*
	N = 248	N = 656	
	Mean ± SD	Mean ± SD	
	n (%)	n (%)	
Birth weight (g)	3140.97 ± 29.35	3165.19 ± 17.71	0.476
5-min Apgar score < 7	3 (1.2)	8 (1.2)	NS
Admission to neonatal intensive care unit	23 (9.27)	46 (6.86)	NS
Maternal and fetal infections	0 (0)	2 (0.30)	NS

	Dinoprostone	Misoprostol	
	N = 238	N = 639	
Fetal acidosis: (pH < 7.15 and/or lactate > 5 mmol/L)	81 (34.03)	135 (21.12)	***0.0007***
Severe fetal acidosis: (pH < 7 and/or lactate > 8 mmol/L	13 (5.46)	20 (3.13)	0.11

NS = Non-significant. Significant values are in bolditalics.

There was no significant difference between the two groups in fetal weight or in 5-min Apgar scores < 7. The rate of fetal acidosis (umbilical arterial pH < 7.15 and/or lactate levels > 5 mmol/L) was higher in the dinoprostone group than the misoprostol group (*p* = 0.0007). There was no significant between-group difference in severe fetal acidosis (umbilical arterial pH < 7 and/or lactate levels > 8 mmol/L). We had no pH or lactate values for 27 newborns (3%), 10 in the dinoprostone group and 17 in the misoprostol group.

There was no significant between-group difference in transfer to neonatal intensive care or maternal–fetal infection. No newborn needed hypothermia for brain protection and there was no fetal death during delivery or the immediate post-partum period.

## Discussion

The first drugs used for the induction of labor in PROM were oxytocin and prostaglandin E2^9,10^. After 37 WG, the American College of Obstetricians and Gynecologists (ACOG) recommends the use of oxytocin for induction of labor in PROM^5^. The National College of French Obstetricians Gynecologists (CNGOF) recommends the use of prostaglandins first line for an unfavorable cervix, as do the Royal College of Obstetricians and Gynaecologists (RCOG) and the National Institute for Health and Care Excellence (NICE) in the UK^6^.

Because of its off-label use in obstetrics, misoprostol has been widely studied and compared with oxytocin, mechanical methods or placebos. All studies have concluded that misoprostol does not increase cesarean section rates or fetal or maternal morbidity^6,11–13^. However, few studies have compared the use of misoprostol and prostaglandin E2 for the induction of labor in PROM.

Our study, although retrospective, had a large number of patients in comparison with other literature reports. The rate of vaginal delivery was greater with dinoprostone for induction of labor than with misoprostol (88.7% vs. 82.3%; *p* = 0.016). To date, no other study has reported this result, though Ayad et al. reported a finding at the limit of significance^14^ for induction of labor in patients with PROM (Table 8). In a recent study, Mlodawski et al. compared misoprostol vaginal insert versus dinoprostone vaginal insert for the induction of labor with intact membranes. Their results are in accordance with ours and indicate an increased risk of cesarean section with vaginal misoprostol (OR 2.71 95% CI 1.63–4.47)^15^. In recent years, several studies have focused on the induction of labor with misoprostol, but few have studied the specific case of PROM. The review by Kerr et al. published in 2021, included thirteen randomized trials that compared low-dose oral misoprostol with the prostaglandin dinoprostone administered vaginally^16^. They reported that the use of oral misoprostol probably results in fewer cesarean sections than vaginal dinoprostone (RR 0.84, 95% CI 0.78–0.90; 13 trials, 9676 women; evidence of moderate uncertainty). However, they concluded that the majority of trials included women with both intact and ruptured membranes, making meaningful analyses very difficult and that the subgroups were very unbalanced^16^.

**Table 8:** Comparative studies of misoprostol and PGE2.

	Study type	Drug used	Mode of delivery
			Vaginal delivery versus Cesarean section
Frohn et al. 2002^17^	Prospective	Misoprostol 50 µg	No difference
	109 Patients	PGE2 gel 2.5 mg	
Ayad et al. 2002^14^	Prospective	Misoprostol 50 µg	No difference
	238 Patients	PGE2 gel 0.5 mg	*p* = 0.058
Chaudhuri et al. 2011^18^	Prospective	Misoprostol 25 µg	No difference
	212 Patients	PGE2 gel 0.5 mg	*p* = 0.10
Nagpal et al. 2009^19^	Prospective	Misoprostol 50 µg	No difference
	61 Patients	PGE2 gel 0.5 mg	
Abraham et al. 2014^20^	Retrospective	Misoprostol 50 µg	No difference
	98 Patients	Dinoprostone 10 mg	
Zhang et al. 2015^21^	4 Prospective studies	Meta-analysis	No difference
	615 Patients		
Sire et al. 2019	Retrospective	Misoprostol 50 µg	Increased rate of cesarean section with misoprostol
	904 patients	Dinoprostone 10 mg	*p* = 0.016

In our study, the rate of cesarean section was 17.7% in the misoprostol group. This corresponds to the average rate in our maternity unit, but is lower than the French average of 20.4% published by the National Institute of Health and Medical Research (INSERM) in 2016^22^. Our rate of cesarean section can be explained by faster labor and hence a higher risk of abnormal fetal heart rate, due to uterine hyperstimulation. In the misoprostol group, there was a higher risk of uterine tachysystole, albeit non-significant. In the dinoprostone group, there was a significant increase in the risk of uterine hypertonicity, but this effect was reversible upon removal of the dinoprostone vaginal insert. This was confirmed by the indications for cesarean section, as there was a significant between-group difference, with a higher rate of cesarean section for abnormal fetal heart rate in the misoprostol group (65.5% vs. 50%; *p* = 0.005), without increased fetal acidosis at birth in the sub-group of cesarean sections.

In their 2018 randomized study in 270 patients^1^, Pourali et al. compared intravenous oxytocin and sublingual misoprostol (25 µg every 4 h). The side effects due to uterine contractions in the misoprostol group were tachysystole (14.2%), uterine hypertonicity (4.2%), and abnormal fetal heart during tachysystole or uterine hypertonicity (10%). In the oxytocin group, these side effects were present in 5.8%, 4.2% and 8.2% of cases, respectively. There was a statistically significant difference (*p* = 0.023) between the two groups. The rate of cesarean section was the same in the two groups. There was a statistically significant difference (*p* = 0.022) between the two groups in terms of the indication for cesarean section, with more cesarean sections for abnormal fetal heart rate in the misoprostol group. This result correlates with our findings. Other studies have reported tachysystole as a side effect with misoprostol^23–25^.

In a subgroup analysis of the induction of labor between 35 and 37 WG, we found no significant difference between the two groups in the mode of delivery. There is no other such subgroup analysis in the literature.

We found a shorter interval between induction and onset of labor in the misoprostol group (*p* < 0.001), a finding confirmed in the literature^1,11,12^. The duration of active labor was significantly shorter in the misoprostol group (*p* = 0.0023).

We found no statistically significant between-group difference in maternal morbidity, notably hemorrhage during delivery, complications of delivery, and chorioamnionitis during the immediate post-partum period. There were no significant between-group differences in neonatal morbidity in terms of transfer to neonatal intensive care or fetal infection.

Abnormal fetal heart rate was more frequent in the misoprostol group, but the difference was at the limit of significance (*p* = 0.058). It is difficult to interpret these results because of bias in reading fetal heart rhythms (inter- and intra-observer variability), however these results are in accordance with the literature^1^.

We noted more moderate fetal acidosis in the dinoprostone group than in the misoprostol group. The difference was significant, a result not found in the literature. The occurrence of moderate fetal hypoxia in the dinoprostone group may be explained by a longer duration of labor. However, we found no between-group difference in the occurrence of severe hypoxia deleterious for the newborn (*p* = 0.11). Definitions of fetal acidosis vary widely, though severe fetal acidosis is regularly defined by an umbilical arterial pH < 7^26–29^. We found no significant between-group difference in Apgar score at birth, in line with all literature studies comparing misoprostol and prostaglandins^14,18–20^.

The duration of maternal hospitalization after delivery was significantly longer (*p* = 0.006) in the misoprostol group than in the dinoprostone group, which is explained by a higher rate of cesarean section in the misoprostol group. No literature study has reported this difference.

We found no difference in the mode of delivery depending on the dose of misoprostol or dinoprostone. The literature indicates a marked disparity in the mode of administration and dose of misoprostol^13,30–32^.

Meta-analyses of oral^30^ and vaginal^31^ misoprostol drew no conclusions about which was most effective in terms of vaginal delivery rate and minimizing side effects. Vaginal misoprostol remained in the blood longer than oral misoprostol. The same result was found for rectal misoprostol, except that plasma concentrations at 240 min were lower than with vaginal administration. Oral administration gave a significantly higher serum peak concentration, but a rapid decrease in plasma levels in comparison with vaginal or rectal administration^33,34^. Therefore, the frequency of administration for vaginal misoprostol may be lower. Sublingual misoprostol delivers a higher serum peak concentration than oral misoprostol^32^.

ACOG recommends the administration of 25 µg of vaginal misoprostol every 3–6 h for induction of labor in patients > 37 WG without PROM^35^. Despite its longstanding use, misoprostol (Angusta 25 µg) was only granted a marketing authorization in France in 2018, without any randomized studies proving its effectiveness and safety. The French Higher Health Authority states: “Angusta 25 µg with oral administration has a marketing authorization for the induction of labor. The dose is 25 µg every two hours or 50 µg every 4 h. The maximum dose is 200 µg over 24 h”^36^.

The literature data indicate that misoprostol does not increase maternal and fetal morbidity. Our findings, on the other hand, indicate an increased rate of cesarean section with repeated use of vaginal misoprostol 50 µg. It is reasonable to think that a lower dose of misoprostol (25 µg versus 50 µg) may reduce tachysystole or hypertonia (as shown in a literature review^37^) and thereby reduce the rate of cesarean section due to fetal heart rate abnormalities observed in our study. Oral administration may reduce abnormal fetal heart rate.

Our study, with its large number of participants compared with other studies published to date, has biases, in particular because it is retrospective and compares two drugs used at two different periods. It is therefore necessary to standardize the dose and route of administration of misoprostol in a randomized and comparative clinical trial in the specific case of PROM.

## Conclusion

Our study reveals a significant increase in the risk of cesarean section when using repeat 50 µg doses of vaginal misoprostol in comparison with 10 mg of vaginal dinoprostone in the induction of labor in PROM after 35 WG in women with an unfavorable cervix. This can be explained by abnormal fetal heart rate and abnormal uterine contractility. There was no increase in fetal or maternal morbidity with either drug.

## Data Availability

On request.

## References

[1] Pourali L, Saghafi N, Eslami Hasan Abadi S, Tara F, Vatanchi AM, Motamedi E (2018). Induction of labour in term premature rupture of membranes; oxytocin versus sublingual misoprostol; a randomised clinical trial. J. Obstet. Gynaecol..

[2] Schmitz T (2018). Preterm premature rupture of membranes: CNGOF Guidelines for clinical practice: Short version. Gynecol. Obstet. Fertil. Senol..

[3] Declenchement artificiel du travail a partir de 37 semaines d’amenorrhee. Rev Sage-Femme. **8**(1), 53–56 (2009).

[4] Couteau C, Haumonté J-B, Bretelle F, Capelle M, D’Ercole C (2013). Management of preterm and prelabour rupture of membranes in France. J. Gynecol. Obstet. Biol. Reprod. (Paris).

[5] ACOG Practice Bulletin No 188 (2018). Prelabor rupture of membranes. Obstet. Gynecol..

[6] 1 Guidance | Inducing labour | Guidance | NICE [Internet]. [Retrieved Sept 5, 2019]. Available at: https://www.nice.org.uk/guidance/cg70/chapter/1-Guidance#induction-of-labour-in-specific-circumstances

[7] Communiqués du CNGOF [Internet]. [Retrieved Feb 6, 2019]. Available at: http://www.cngof.fr/patientes/presse/474-communiques-du-cngof

[8] Ayres-de-Campos D, Arulkumaran S (2015). FIGO consensus guidelines on intrapartum fetal monitoring: Introduction. Int. J. Gynecol. Obstet..

[9] Carbonne B, Goffinet F, Cabrol D (1996). Vaginal administration prostaglandin E2 in premature ruptured membranes at term with an unfavorable cervix. J. Gynecol. Obstet. Biol. Reprod. (Paris).

[10] Hannah ME (1996). Induction of labor compared with expectant management for prelabor rupture of the membranes at term: TERMPROM Study Group. N. Engl. J. Med..

[11] Ngai SW, To WK, Lao T, Ho PC (1996). Cervical priming with oral misoprostol in pre-labor rupture of membranes at term. Obstet. Gynecol..

[12] Levy R, Vaisbuch E, Furman B, Brown D, Volach V, Hagay ZJ (2007). Induction of labor with oral misoprostol for premature rupture of membranes at term in women with unfavorable cervix: A randomized, double-blind, placebo-controlled trial. J. Perinat. Med..

[13] Lin MG, Nuthalapaty FS, Carver AR, Case AS, Ramsey PS (2005). Misoprostol for labor induction in women with term premature rupture of membranes: A meta-analysis. Obstet Gynecol..

[14] Ayad IAA (2002). Vaginal misoprostol in managing premature rupture of membranes. East Mediterr. Health J. Rev. Sante Mediterr Orient Al-Majallah Al-Sihhiyah Li-Sharq Al-Mutawassit..

[15] Mlodawski J, Mlodawska M, Armanska J, Swiercz G, Gluszek S (2021). Misoprostol vs dinoprostone vaginal insert in labour induction: Comparison of obstetrical outcome. Sci. Rep..

[16] Kerr RS, Kumar N, Williams MJ, Cuthbert A, Aflaifel N, Haas DM, Weeks AD (2021). Low-dose oral misoprostol for induction of labour. Cochrane Database Syst. Rev..

[17] Frohn WE, Simmons S, Carlan SJ (2002). Prostaglandin E2 gel versus misoprostol for cervical ripening in patients with premature rupture of membranes after 34 weeks. Obstet. Gynecol..

[18] Chaudhuri S, Mitra SN, Banerjee PK, Biswas PK, Bhattacharyya S (2011). Comparison of vaginal misoprostol tablets and prostaglandin E2 gel for the induction of labor in premature rupture of membranes at term: A randomized comparative trial. J. Obstet. Gynaecol. Res..

[19] Nagpal MB, Raghunandan C, Saili A (2009). Oral misoprostol versus intracervical prostaglandin E2 gel for active management of premature rupture of membranes at term. Int. J. Gynaecol. Obstet..

[20] Abraham C, Meirowitz N, Kohn N (2014). Labor induction for premature rupture of membranes using vaginal misoprostol versus dinoprostone vaginal insert. Am. J. Perinatol..

[21] Zhang Y, Wang J, Yu Y, Xie C, Xiao M, Ren L (2015). Misoprostol versus prostaglandin E2 gel for labor induction in premature rupture of membranes after 34 weeks of pregnancy. Int. J. Gynaecol. Obstet..

[22] La santé des mères et des nouveau-nés: premiers résultats de l’enquête nationale périnatale 2016 [Internet]. Salle de presse | Inserm. 2017 [Retrieved March 31, 2019]. Available at: https://presse.inserm.fr/la-sante-des-meres-et-des-nouveau-nes-premiers-resultats-de-lenquete-nationale-perinatale-2016/29668/

[23] Sanchez-Ramos L, Chen AH, Kaunitz AM, Gaudier FL, Delke I (1997). Labor induction with intravaginal misoprostol in term premature rupture of membranes: A randomized study. Obstet. Gynecol..

[24] Wing DA, Paul RH (1998). Induction of labor with misoprostol for premature rupture of membranes beyond thirty-six weeks’ gestation. Am. J. Obstet. Gynecol..

[25] Khoury AN, Zhou QP, Gorenberg DM, Nies BM, Manley GE, Mecklenburg FE (2001). A comparison of intermittent vaginal administration of two different doses of misoprostol suppositories with continuous dinoprostone for cervical ripening and labor induction. J. Matern. Fetal. Med..

[26] Gjerris AC, Staer-Jensen J, Jørgensen JS, Bergholt T, Nickelsen C (2008). Umbilical cord blood lactate: A valuable tool in the assessment of fetal metabolic acidosis. Eur. J. Obstet. Gynecol. Reprod. Biol..

[27] Tuuli MG, Stout MJ, Shanks A, Odibo AO, Macones GA, Cahill AG (2014). Umbilical cord arterial lactate compared with pH for predicting neonatal morbidity at term. Obstet. Gynecol..

[28] Knutzen L, Anderson-Knight H, Svirko E, Impey L (2018). Umbilical cord arterial base deficit and arterial pH as predictors of adverse outcomes among term neonates. Int. J. Gynaecol. Obstet..

[29] Allanson ER, Waqar T, White C, Tunçalp Ö, Dickinson JE (2017). Umbilical lactate as a measure of acidosis and predictor of neonatal risk: A systematic review. BJOG Int. J. Obstet. Gynaecol..

[30] Alfirevic Z, Aflaifel N, Weeks A (2014). Oral misoprostol for induction of labour. Cochrane Database Syst Rev..

[31] Hofmeyr GJ, Gülmezoglu AM, Pileggi C (2010). Vaginal misoprostol for cervical ripening and induction of labour. Cochrane Database Syst. Rev..

[32] Tang OS, Schweer H, Seyberth HW, Lee SWH, Ho PC (2002). Pharmacokinetics of different routes of administration of misoprostol. Hum. Reprod. Oxf. Engl..

[33] Khan R-U, El-Refaey H, Sharma S, Sooranna D, Stafford M (2004). Oral, rectal, and vaginal pharmacokinetics of misoprostol. Obstet. Gynecol..

[34] Zieman M, Fong SK, Benowitz NL, Banskter D, Darney PD (1997). Absorption kinetics of misoprostol with oral or vaginal administration. Obstet. Gynecol..

[35] Wing DA, Gaffaney CAL (2006). Vaginal misoprostol administration for cervical ripening and labor induction. Clin. Obstet. Gynecol..

[36] Haute Autorité de Santé - ANGUSTA 25 µg (misoprostol), par voie orale, utérotonique [Internet]. [Retrieved March 7, 2019].Available at: https://www.has-sante.fr/portail/jcms/c_2862078/fr/angusta-25-g-misoprostol-par-voie-orale-uterotonique

[37] McMaster K, Sanchez-Ramos L, Kaunitz A (2015). Balancing the efficacy and safety of misoprostol: A meta-analysis comparing 25 versus 50 micrograms of intravaginal misoprostol for the induction of labour. BJOG Int. J. Obstet. Gynaecol..

